# Langerhans Cell Histiocytosis Involving the Hypothalamic–Pituitary System Accompanied by Adipsic Diabetes Insipidus and Multiple Anterior Pituitary Hormone Deficiencies: A Case Report

**DOI:** 10.3390/jcm15207877

**Published:** 2026-10-13

**Authors:** Masakazu Uejima, Yukako Kurematsu, Kuniaki Ozaki, Kosuke Kaji, Kei Moriya, Tadashi Namisaki, Akira Mitoro, Hitoshi Yoshiji

**Affiliations:** 1 Department of Gastroenterology, Nara Medical University, Kashihara 634-8521, Nara, Japan; kajik@naramed-u.ac.jp (K.K.); moriyak@naramed-u.ac.jp (K.M.); tadashin@naramed-u.ac.jp (T.N.); mitoroak@naramed-u.ac.jp (A.M.); yoshijih@naramed-u.ac.jp (H.Y.); 2 Department of Diabetes and Endocrinology, Nara Medical University, Kashihara 634-8521, Nara, Japan; yukakok@naramed-u.ac.jp (Y.K.); k-ozaki@naramed-u.ac.jp (K.O.)

**Keywords:** Langerhans cell histiocytosis, adipsic diabetes insipidus, multiple anterior pituitary hormone deficiencies, hypothalamic–pituitary system involvement, hypothalamic syndrome

## Abstract

Adult-onset Langerhans cell histiocytosis (LCH) is an uncommon disease. Central nervous system involvement, particularly of the hypothalamic–pituitary complex, can provoke severe endocrine and neurological complications. We report the case of a 39-year-old female patient with a history of pulmonary LCH who developed central diabetes insipidus (CDI), anterior pituitary dysfunction, and severe hypothalamic syndrome, accompanied by adipsia, thermoregulatory dysfunction, and cognitive impairment. Brain magnetic resonance imaging revealed a hypothalamic mass with peritumoral edema. Following ineffective corticosteroid therapy, cladribine was administered. Although the tumor size and edema significantly decreased, it caused prolonged grade 4 bone marrow suppression, necessitating treatment discontinuation. Despite the marked radiological improvement, multiple anterior pituitary hormone deficiencies, cognitive impairment, and irreversible hypothalamic dysfunction persisted. Furthermore, concomitant adipsic CDI and thermoregulatory failure provoked severe dehydration, complicating clinical management. Ultimately, the patient died of sudden cardiac arrest, secondary to extreme hypernatremia (170 mEq/L) and dehydration-induced cardiopulmonary collapse. The case presented herein highlights the possible devastating consequences of hypothalamic involvement in adult multisystem LCH. While cladribine is effective at reducing the tumor burden, it fails to reverse established neuroendocrine and neurodegenerative damage. Early therapeutic and further lifelong interventions, specifically for adipsic CDI, are crucial to preventing fatal outcomes and maintaining quality of life.

## 1. Introduction

Adult-onset Langerhans cell histiocytosis (LCH) is a rare disorder characterized by abnormal clonal dendritic cells that affect multiple organs, including those of the endocrine system. The annual incidence of LCH is 1–2 cases per million, with a similar rate for both sexes [1]. LCH is classified into single-system LCH, which affects only one organ or system, or multisystem LCH, which affects two or more organs or systems. Autopsies of LCH cases indicate central nervous system (CNS) involvement, especially of the hypothalamic–pituitary system (HPS), with the incidence ranging widely from 5% to 50% [2]. Central diabetes insipidus (CDI) is a frequent manifestation of multisystem LCH with HPS invasion, occurring in 30% of cases [1]. Characteristically, LCH disseminates to the hypothalamus several years after CDI onset [1], facilitating the development of anterior pituitary dysfunction (APD). APD occurs in two-thirds of cases of LCH with CDI, causing deficiencies in growth hormone in 53–67% and in gonadotropin in 55–58% [2]. Hypothalamic LCH also affects autonomic nervous system centers, causing hypothalamic syndrome manifestations such as lack of thirst, thermoregulatory dysfunction, and hyperphagia. Moreover, hypothalamic LCH can sometimes present with neuropsychological disorders, such as cognitive impairment and hypothalamic amnesia [2], and neurological symptoms that may result not only from the mass effect of a lesion but also from the neurodegenerative effects that LCH itself exerts on the CNS [3].

Here, we describe the case of a female patient with multisystem LCH with pulmonary and HPS involvement, complicated by APD, adipsic CDI, and hypothalamic syndrome.

## 2. Case Presentation

**Figure 1:**
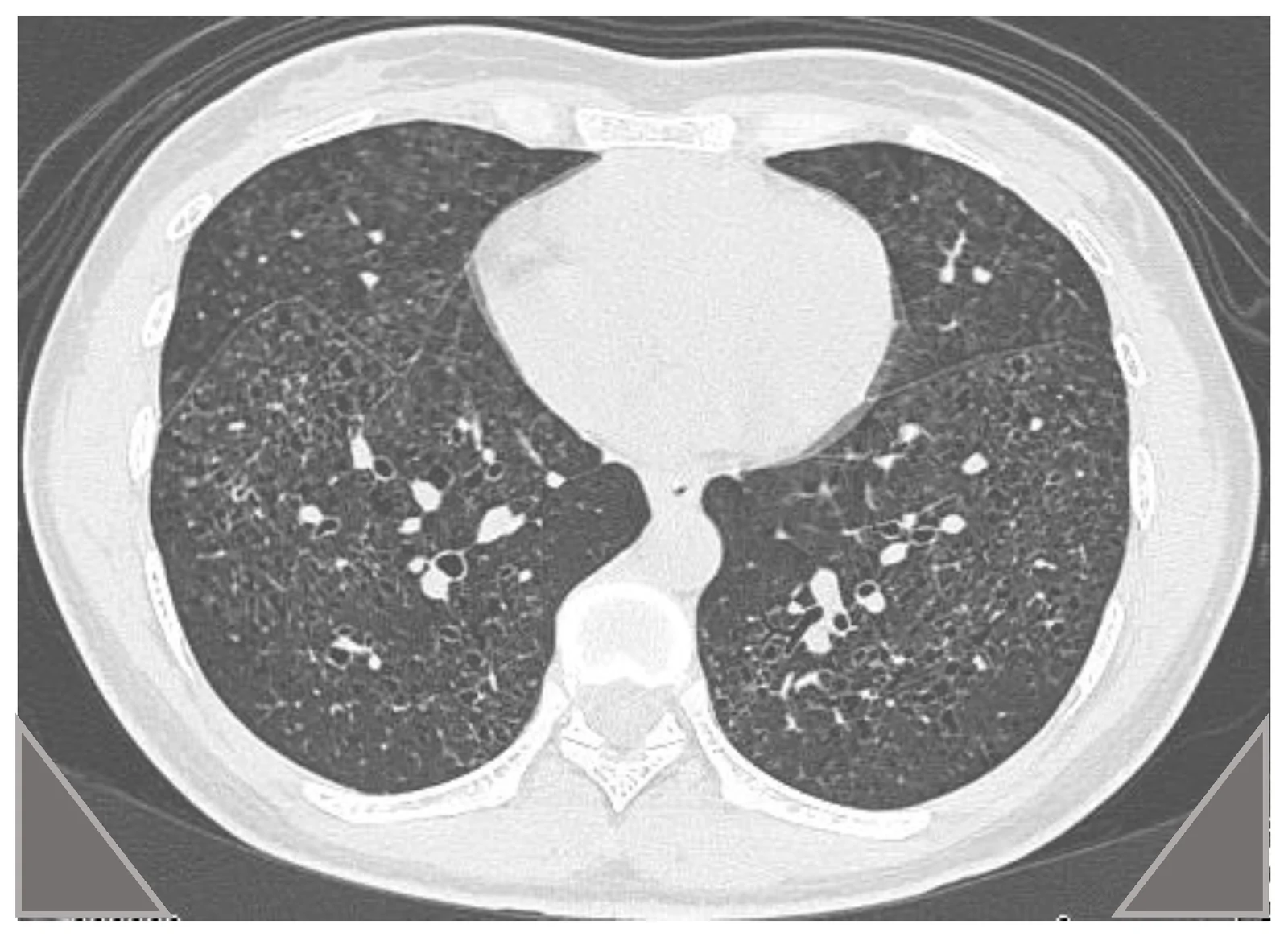
Chest computed tomography revealed ground-glass nodules and cysts in a nodulocystic pattern, characteristic of pulmonary LCH.

**Figure 2:**
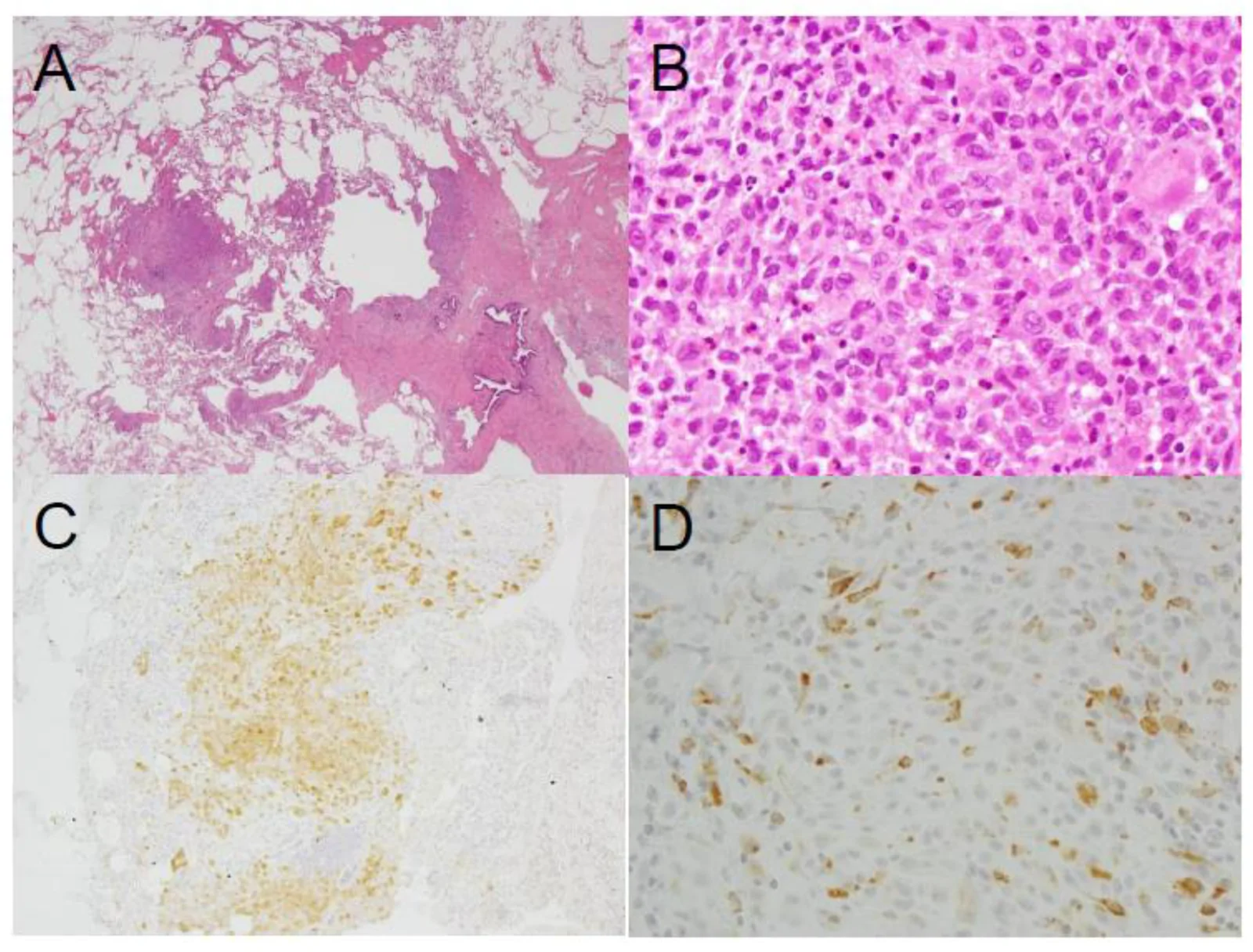
Lung biopsy specimen, obtained via video-assisted thoracic surgery. Hematoxylin and eosin staining at (**A**) 40× and (**B**) 400×; (**C**) 40× S-100 staining; and (**D**) 400× CD68 staining, consistent with pulmonary Langerhans cell histiocytosis.

**Figure 3:**
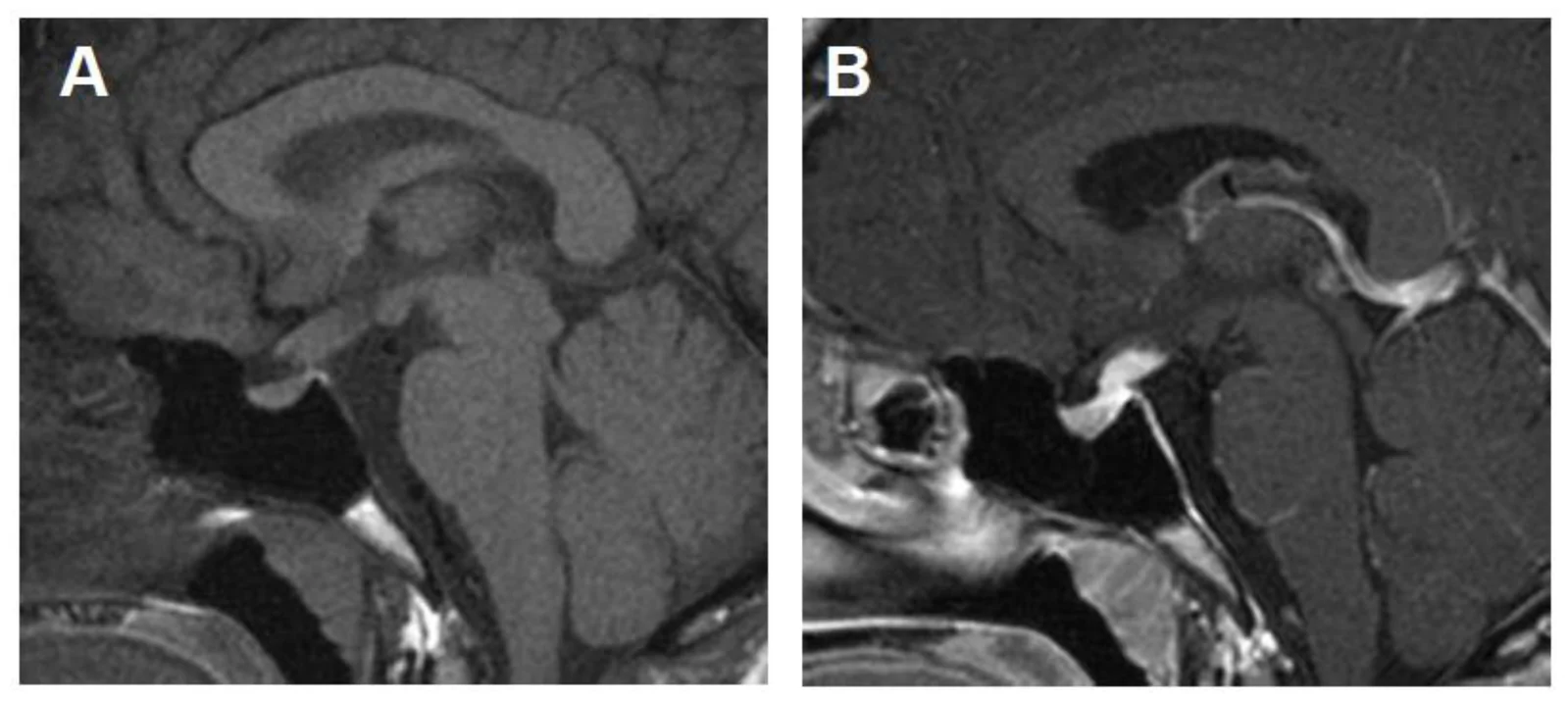
Brain magnetic resonance imaging (MRI) at age 34, when diabetes insipidus emerged. Pituitary MRI revealed the disappearance of T1-weighted high-intensity signal in the (**A**) posterior pituitary lobe and the thickened pituitary stalk on the (**B**) post-contrast image.

The case involved a female patient who passed away at age 39 (in 2012), and the patient was first referred to our hospital at the age of 30 (in 2002) because of previously noted diffuse reticulonodular infiltrates on a chest radiograph. Chest computed tomography (CT) revealed ground-glass nodules and cysts in a nodulocystic pattern characteristic of pulmonary LCH (Figure 1). Thoracoscopic lung biopsy was performed, and histopathological findings were indicative of pulmonary LCH (Figure 2). The patient, who smoked 10 cigarettes a day at that time, was advised to quit smoking; however, the patient could not. At ages 31 and 32 (in 2003–2004), the patient developed pneumothorax, provoked by LCH. At age 34 (in 2006), polydipsia, polyuria, galactorrhea, and amenorrhea emerged. Brain magnetic resonance imaging (MRI) revealed a thickened pituitary stalk and the disappearance of a T1-weighted high-intensity signal at the posterior pituitary lobe (Figure 3).

Endocrinological examination revealed decreased levels of antidiuretic hormone (ADH), 0.2 pg/mL (0.0–3.1); and plasma osmolar pressure (Posm), 298 mOsm/L (275–290); and elevated prolactin (PRL), 78.2 ng/mL (4.3–13.7), indicating a pituitary stalk lesion, and luteinizing hormone (LH), 2.17 IU/L (1.71–8.59); follicle-stimulating hormone (FSH), 5.84 U/L (1.49–12.40); and estradiol, 10.4 pg/mL (28.8–196.8), indicative of central hypogonadism. Other pituitary hormones, including growth hormone (GH)/insulin-like growth factor 1 (IGF-1), adrenocorticotropic hormone (ACTH)/cortisol, and thyroid-stimulating hormone (TSH)/thyroxine were within their normal ranges (Table 1). Prednisolone (PSL) was initiated at 30 mg/day, followed by steroid pulse therapy, due to suspected lymphocytic infundibulo-neurohypophysitis based on the patient’s age and sex, as well as on MRI findings of pituitary stalk thickening and loss of the posterior pituitary bright spot on T1-weighted images. However, no changes were observed in clinical symptom manifestations or imaging findings, and the treatment was tapered and discontinued early; subsequently, the patient was placed under outpatient follow-up after the initiation of transnasal desmopressin (ddAVP).

**Figure 4:**
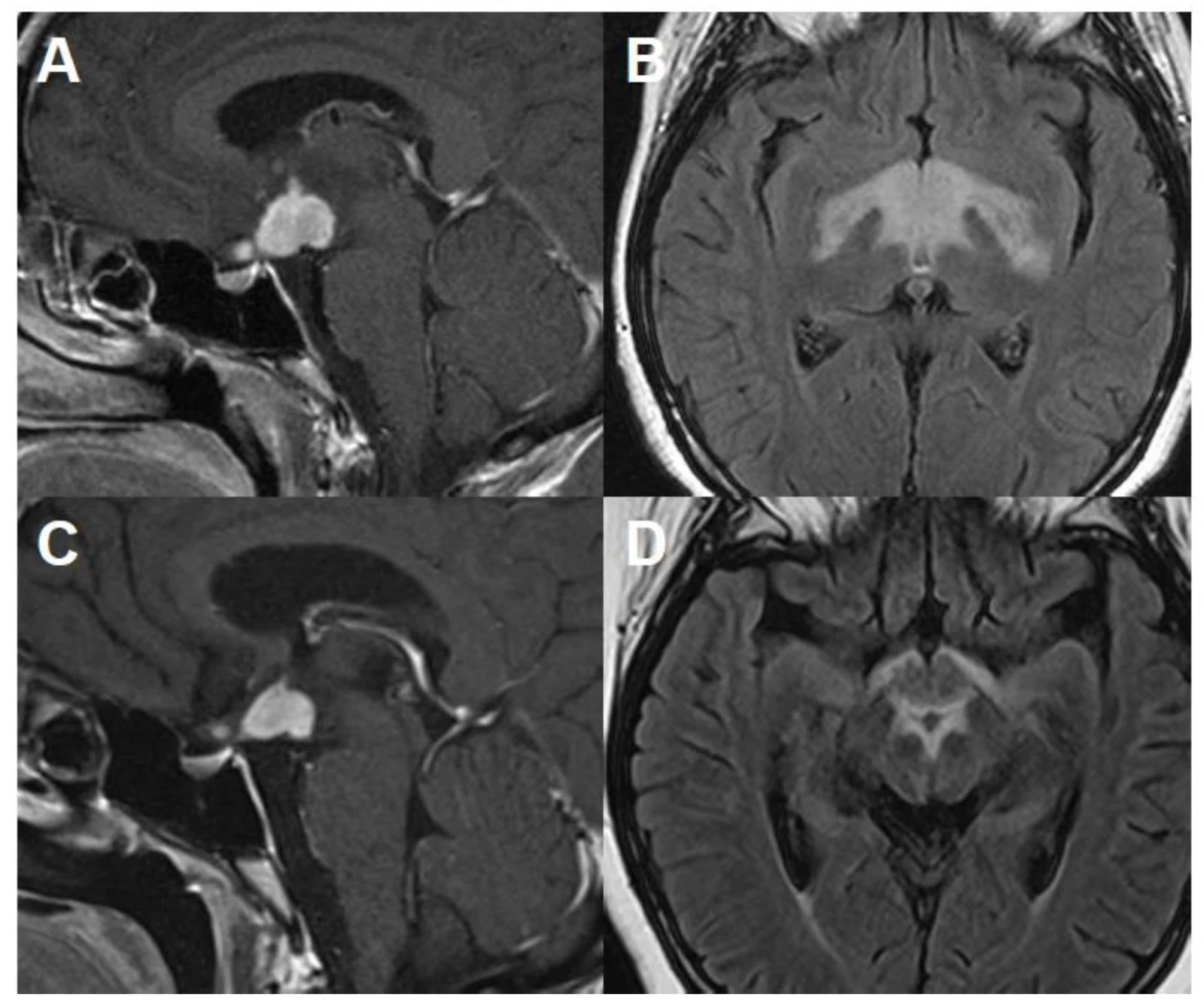
Brain magnetic resonance imaging (MRI) (**A**,**B**) before prednisolone (PSL) therapy and (**C**,**D**) when PSL was tapered to 20 mg per day. (**A**) A 25 mm hypothalamic mass was noted on the post-contrast image. (**B**) Peritumoral edema around the hypothalamus and basal ganglia, showing fluid-attenuated inversion recovery (FLAIR) hyperintensity. (**C**) Minimal shrinkage of the hypothalamic mass was observed on the post-contrast image. (**D**) Peritumoral edema showed remarkable improvement on FLAIR.

**Figure 5:**
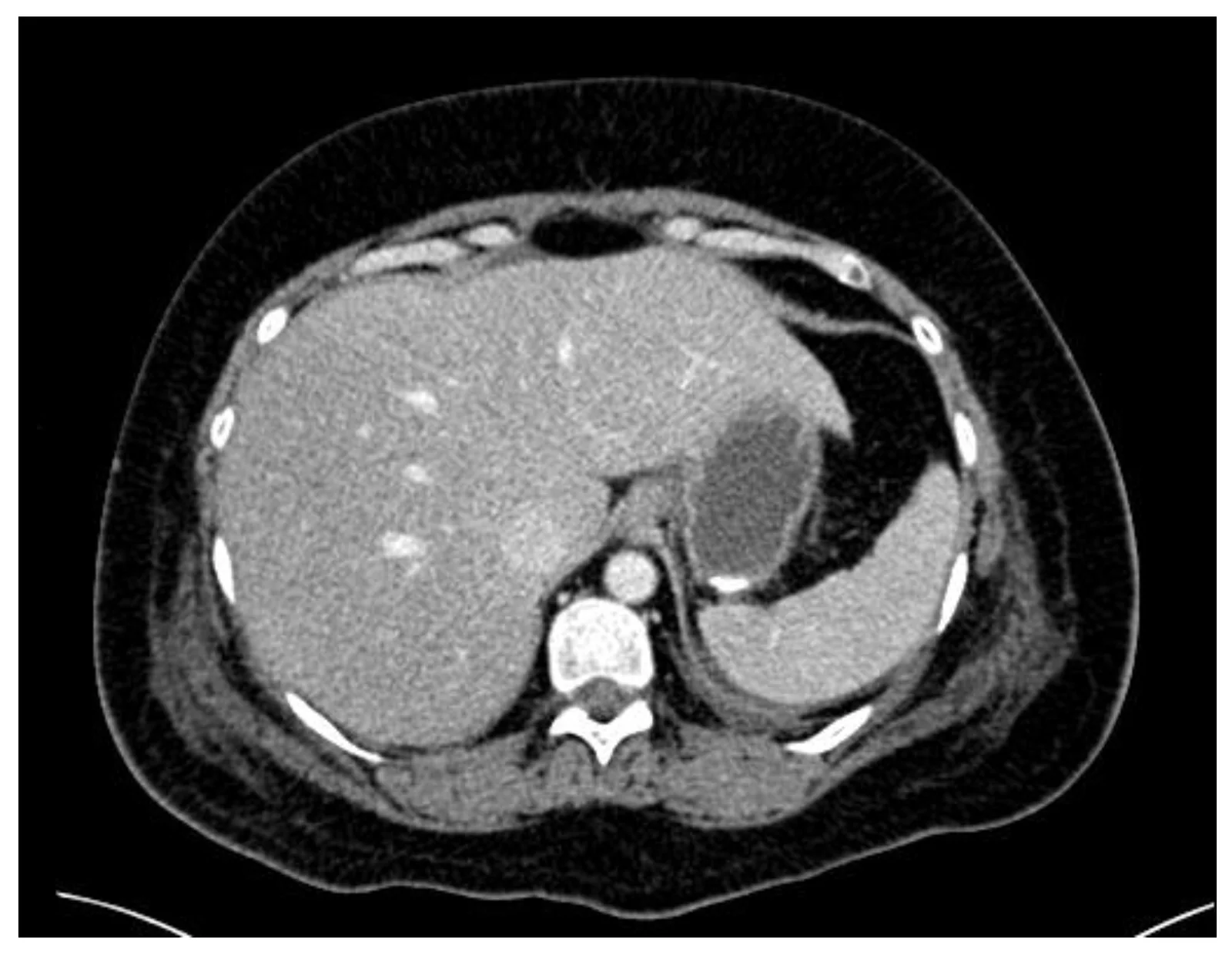
Contrast-enhanced abdominal computed tomography showed no hepatic or splenic infiltration by LCH.

**Figure 6:**
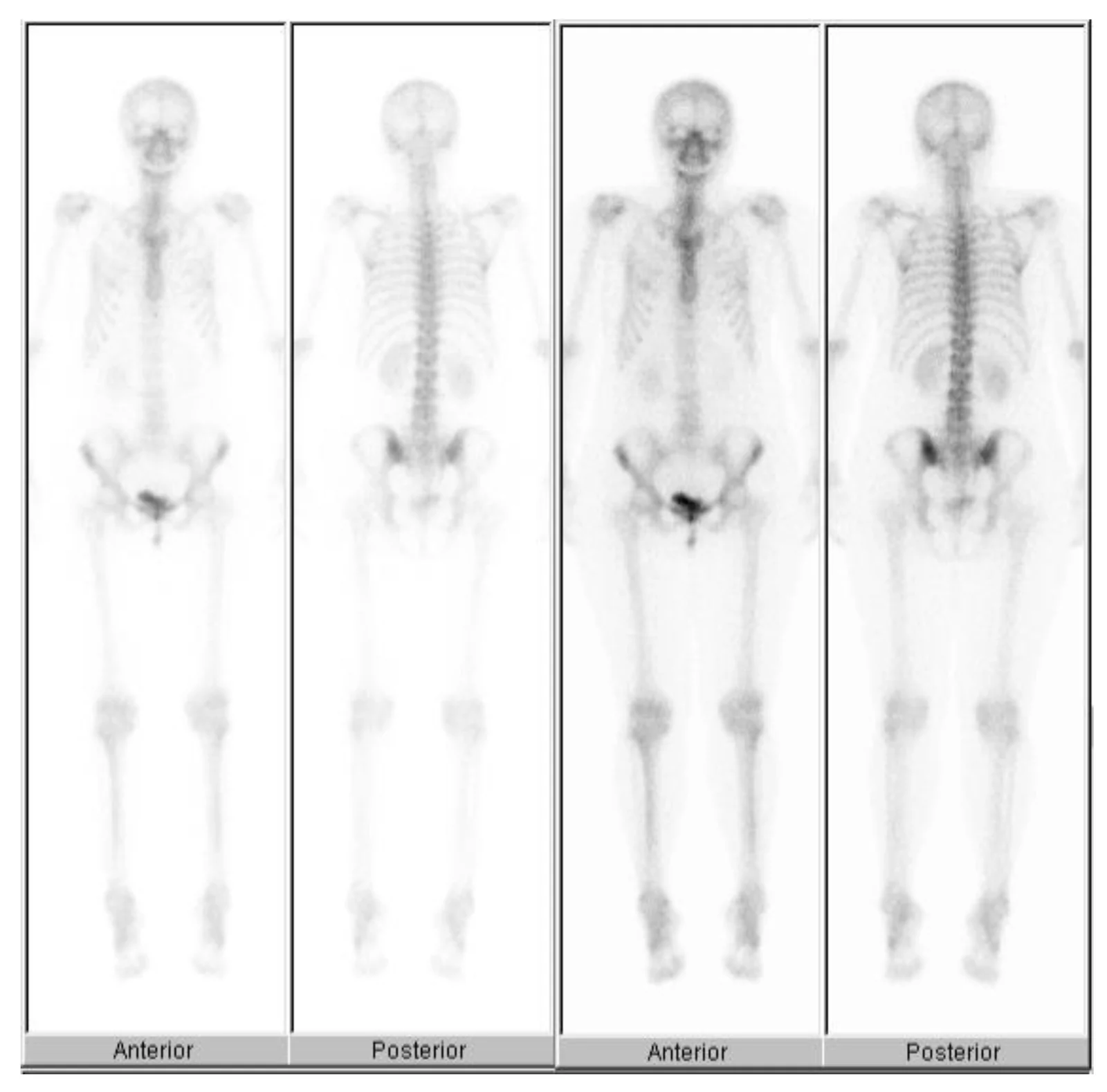
Bone scintigraphy showed no evidence of osseous LCH involvement.

At age 35 (in 2007), the patient developed a 38 °C fever, significant weight gain, and general fatigue; simultaneously, speech and behavioral abnormalities, as well as impaired memory, became evident and began to interfere with the patient’s daily life. Consequently, the patient was hospitalized for further diagnostic evaluation and treatment, at which time the patient’s physical characteristics were as follows: height, 162 cm; weight, 78 kg (body surface area: 1.8 square meters); blood pressure, 124/80 mmHg; and body temperature, 38.1 °C. The patient was alert and oriented. Neurological examination revealed no abnormalities in the cranial nerves or in motor or sensory systems; however, the patient scored 24 on the revised version of Hasegawa’s Dementia Scale (HDS-R) and was unable to correctly identify the day of the week. Endocrinological examination (Table 2) was indicative of central hypothyroidism (TSH 1.07 mU/L, free thyroxine 0.70 ng/dL, free triiodothyronine 2.2 pg/mL) and central hypoadrenalism (ACTH 5.4 pg/mL, cortisol 0.8 μg/dL). A provocation test using corticotropin-releasing hormone, thyrotropin-releasing hormone, gonadotropin-releasing hormone, and growth hormone-releasing factor demonstrated blunted responses in ACTH, LH, and FSH (Table 3); the hypertonic saline test showed no response in ADH, suggesting CDI (Table 4); brain MRI revealed a 25 mm mass lesion in the hypothalamus, accompanied by peritumoral edema (Figure 4A,B). To reevaluate the diagnosis, we recommended a biopsy of the hypothalamic lesion, but the patient declined; accordingly, the brain lesion was diagnosed clinically as LCH with hypothalamic–pituitary involvement, without histologic confirmation, based on her history of pulmonary LCH and compatible MRI findings. Contrast-enhanced abdominal CT (Figure 5) and bone scintigraphy (Figure 6), performed to assess additional systemic involvement, revealed no hepatic or bone lesions, respectively. PSL was initiated at 60 mg per day, and then gradually tapered to 20 mg daily; subsequent brain MRI revealed a smaller peritumoral edematous change, but the overall effect on hypothalamic shrinkage was minimal (Figure 4C,D); moreover, the appearance of iatrogenic Cushing’s syndrome signs led to tapering and eventual discontinuation of PSL and switching to hydrocortisone 20 mg per day as replacement therapy. For replacement therapy, levothyroxine was also initiated and maintained at 75 μg per day. Although strongly recommended, the patient did not agree to sex hormone replacement therapy, owing to concerns regarding menstrual management; almost simultaneously, hypothalamic dysfunction manifestations, such as thermoregulation disorder, hyperphagia, and adipsia, became more apparent. To manage adipsic CDI, we set her target body weight at 75 kg and advised her and her husband to monitor her weight several times daily, ensure a daily water intake of at least 1,500 mL, and administer ddAVP twice daily (2.5 μg in the morning and 5 μg at night), with an additional 2.5 μg if her weight fell below 75 kg.

**Figure 7:**
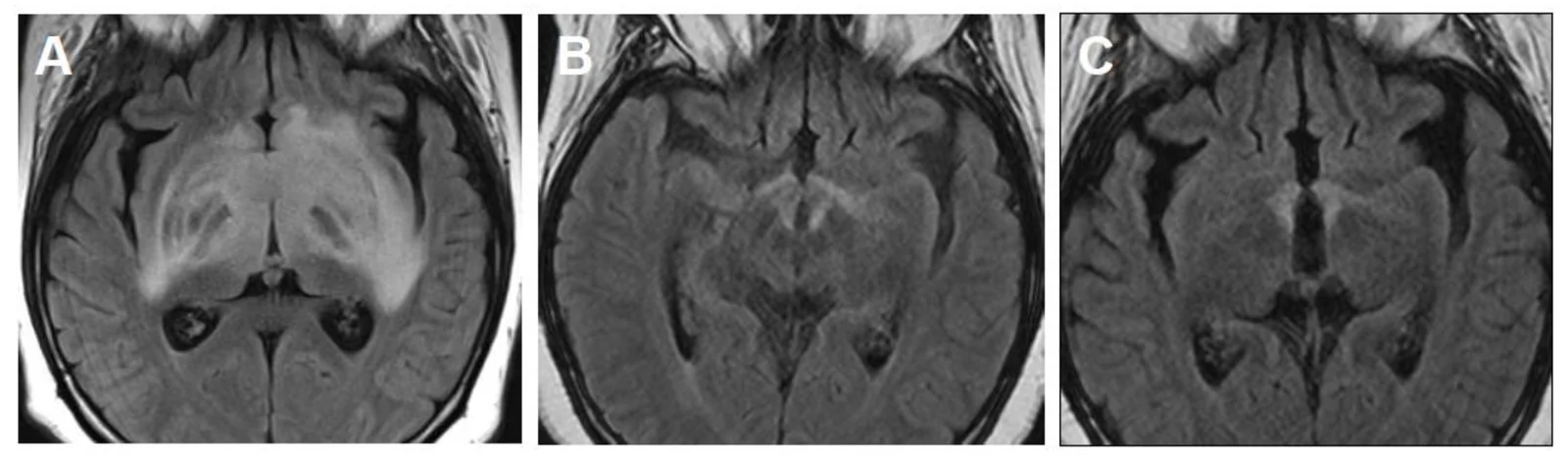
Brain magnetic resonance imaging (MRI) before and after 2-CdA (cladribine) therapy on fluid-attenuated inversion recovery (FLAIR). (**A**) Brain MRI during the patient’s admission to the emergency room, with marked worsening of the peritumoral edema around the hypothalamus. (**B**) Brain MRI after two courses of 2-CdA administration; peritumoral edema and hypothalamic mass remarkably ameliorated and almost disappeared. (**C**) Brain MRI 2 years after 2-CdA therapy, showing no worsening of the hypothalamic lesion or peritumoral edema compared with (**B**).

At age 36 (in 2008), the patient was referred to the emergency department for severe headaches. Brain MRI showed a worsening hypothalamic mass and peritumoral edema (Figure 7A), resulting in the patient’s hospitalization for elevated intracranial pressure. Following glycerol injections, as a primary course of chemotherapy, 5 mg per body (about 3 mg per square) of 2-CdA (cladribine) was intravenously administered for 3 days, which led to prolonged grade 4 bone marrow suppression, requiring red blood cell and platelet transfusion, as well as subcutaneous injection of filgrastim. After a 2-month interval, 4 mg per body of 2-CdA was intravenously administered again for 3 days; however, grade 4 bone marrow suppression recurred; therefore, 2-CdA therapy was discontinued. However, the imaging results were remarkable: MRI after the two courses of 2-CdA showed alleviation of peritumoral edema and significant shrinkage of the hypothalamic mass (Figure 7B). Despite the improvement in MRI findings, irreversible functional damage to the hypothalamus was sustained. Because of thermoregulatory dysfunction, body temperature fluctuated between 32 °C and 40 °C with the ambient temperature; furthermore, adipsic CDI caused the loss of the thirst reflex, significantly increasing dehydration risk.

Brain MRI performed at age 38 (in 2010) exhibited no worsening of the hypothalamic lesion (Figure 7C); however, by that time, amnesia and delusions had become severe. Furthermore, because activities of daily living were significantly impaired, the patient became totally dependent on care. Her amnesia and hallucinations made it extremely difficult for her to manage her medications, including ddAVP, or calculate her daily water intake; despite her husband’s devoted caregiving, maintaining her water balance remained difficult, and the patient frequently developed hypo or hypernatremia.

At age 39 (in 2012), the patient was admitted to our hospital with severe generalized fatigue and immobility. Laboratory testing revealed severe hypernatremia (170 mEq/L), consistent with profound dehydration caused by inadequate water intake and insufficient desmopressin administration; despite intravenous fluid replacement, the patient passed away the following day after sudden cardiopulmonary arrest, due to severe hypernatremia-mediated cardiovascular collapse.

## 3. Discussion

LCH develops from clonal multiplication of myeloid precursors that differentiate into CD1a+/CD207+ cells and can involve multiple tissues and organs, including the bone, skin, lungs, and pituitary gland. CNS involvement in LCH typically occurs with multisystem disease and can clinically present with either focal mass lesions or progressive neurodegeneration [2]. The pituitary gland is susceptible to involvement in LCH, particularly the posterior pituitary lobe; the endocrine system, especially the hypothalamic–pituitary axis, is also frequently involved. Pituitary gland involvement occurs in up to 25% of cases, and anterior pituitary deficiencies are present in approximately 20% of cases; this involvement may arise from localized clonal proliferation and immune-mediated inflammatory responses, including the cytokine storm, which contributes to pituitary cell destruction [4].

MRI, with and without gadolinium enhancement, is a useful modality for assessing sellar and suprasellar involvement in LCH. Characteristic findings include infundibular thickening and posterior pituitary bright spot loss, particularly in patients with LCH-related CDI. In addition to suprasellar masses, involvement of the pons, basal ganglia, and white matter may also manifest [2,5]. In the present case, head MRI was critical in the assessment of lesion progression and treatment efficacy, as well as in monitoring the patient’s condition.

In the current adult patient case of multisystem LCH, hypothalamic involvement became clinically evident as CDI several years after the onset of pulmonary disease [1]. Consistent with a previous report [1], this patient developed multiple pituitary hormone deficiencies approximately 2 years after the onset of CDI.

The major concern in LCH and CDI with CNS involvement is the progressive disruption of hypothalamic function, accompanied by cognitive, behavioral, or neuropsychological disturbances. Impaired thirst perception and poor adherence may have clinically significant consequences; weight fluctuations, increased appetite, sleep disturbances, and thermoregulatory dysfunction may also occur. Variability in clinical manifestations and timing of onset underscores the heterogeneity of disease features; moreover, structural abnormalities in the hypothalamic–pituitary axis can indicate broader CNS involvement and associated neurologic sequelae, consistent with the progressive and potentially irreversible nature of this infiltrative disorder [6]. In the present case, amnesia, confabulation, and delusions emerged with disease progression, potentially demonstrating not only the mass effect from the hypothalamic lesion, but also the manifestations of hypothalamic amnesia and limbic disconnection syndrome resulting from the disruption of hypothalamic neural networks.

A review by the Histiocyte Society CNS LCH Study Group identified three neuropathologic patterns of CNS LCH lesions: The first pattern consists of circumscribed granulomas in the brain’s connective tissue spaces; their composition resembles that of LCH granulomas in peripheral organs, with variable numbers of CD1a+ cells and prominent CD8+ T-lymphocytic infiltration, and they preferentially involve circumventricular organs without a blood–brain barrier, such as the pituitary stalk. The second pattern comprises neurodegenerative lesions that primarily affect the cerebellum and brain stem; although CD1a+ cells are absent, marked inflammation dominated by CD8+ lymphocytes is associated with neuronal and axonal degeneration and secondary myelin loss, extensive cases of which lead to atrophy of the cerebellar cortex and white matter. In the third pattern, granulomas from infundibular tumors invade the hypothalamus, with diffuse CD1a+ histiocyte infiltration into the surrounding CNS parenchyma, accompanied by neurodegeneration, which is characterized by marked axonal and neuronal loss in adjacent tissue and intense CD8+ T-lymphocyte-predominant inflammation [5]. Although pathological confirmation was unavailable, the clinical course and MRI findings in our patient were consistent with the first and third patterns of this classification. The tumor-like growth of LCH itself may have caused direct or immune-mediated paraneoplastic inflammation, which could induce irreversible effects on the CNS [7].

Adipsic CDI and loss of thermoregulation significantly complicated disease management in this case; the absence of thirst with an increase in insensible perspiration due to abnormal body temperature increase resulted in extreme dehydration and hypernatremia, which led to cardiovascular collapse and the patient’s death. Survival rates are reportedly excellent in patients without organ dysfunction, but mortality may reach 20% in those with organ dysfunction. Adipsic DI is particularly difficult to manage in patients with cognitive impairment, as its treatment involves twice-daily ddAVP to stabilize urine output and a fixed fluid intake, ideally determined through inpatient observation at diagnosis. Once a baseline eunatremic weight and stable plasma sodium are established, usually with a daily fluid intake of 1.5–2 L, intake can be adjusted for climate, physical activity, and intercurrent illness. Daily weighing is recommended because a sudden decrease from the eunatremic weight may indicate dehydration and the need for additional hypotonic fluid to replace the deficit [8]. In our case, we set her target body weight at 75 kg and advised her and her husband to monitor her weight several times daily, ensure a daily water intake of at least 1500 mL, and administer ddAVP twice daily (2.5 μg in the morning and 5 μg at night), with an additional 2.5 μg if her weight fell below 75 kg; however, prescribing an appropriate fluid regimen was extremely difficult because of the patient’s cognitive and behavioral impairments.

Although treatments for LCH have advanced significantly, disease reactivation rates persist above 30%. Therapy failure is linked to higher risks of mortality and long-term morbidity, as well as LCH-associated neurodegeneration [3]. Multifocal or single brain lesions developing within multisystem disease require chemotherapy, with the most effective being 2-CdA and cytarabine [9]; until recently, reports have described therapeutic efficacy for high-risk, refractory, and recurrent cases, although accompanied by severe toxicity and side effects. In the analysis of 22/23 extrapulmonary adult-onset LCH cases from the French Histiocytosis Group, the overall response rate was 91%, with complete response achieved in 50% (11/22); nine patients (39%) had grade 3–4 neutropenia and/or severe infection [10]. The 2022 international expert consensus recommends cladribine- or cytarabine-based chemotherapy as the first-line treatment for CNS disease [11]. The BRAF V600E mutation occurs in approximately 50% of LCH cases, and next-generation sequencing has shown that BRAF-wild-type cases may also harbor mutations in MAPK-ERK pathway genes. Accordingly, the 2022 International Expert Consensus recommends BRAF V600E screening in all LCH cases. In BRAF-V600-wild-type LCH, next-generation sequencing should be performed to evaluate MAPK-ERK pathway mutations, particularly when the diagnosis is uncertain or second-line treatment is required [11]. Evidence supporting the efficacy of BRAF and MEK inhibitors in both frontline and relapsed settings is emerging. Most reported patients harbored the BRAF V600E mutation and were treated with vemurafenib, whereas MEK inhibitors such as cobimetinib and trametinib have also been described. BRAF and MEK inhibitors provide rapid and near-universal responses, making them preferred therapeutic options for patients requiring prompt reversal of organ compromise [11]. For LCH that is refractory to first-line therapy or associated with end-organ dysfunction (e.g., neurologic impairment or sclerosing cholangitis), alternative conventional regimens or targeted therapy with BRAF or MEK inhibitors should be considered. Although evidence in adults with neurodegenerative disease is limited, BRAF/MEK inhibitor- or cytarabine-based therapy may be favored based on limited pediatric experience [11].

In the present case, when central diabetes insipidus first developed, we diagnosed it as lymphocytic infundibulo-neurohypophysitis based on the patient’s age and sex, as well as MRI findings of pituitary stalk thickening and loss of the posterior pituitary bright spot on T1-weighted images. Steroid pulse therapy, followed by oral prednisolone, was initiated but produced no notable improvement; subsequently, as the hypothalamic lesion worsened, we reassessed the underlying pathology. Based on the patient’s prior history of LCH, the hypothalamic lesion was ultimately diagnosed as LCH, rather than lymphocytic infundibulo-neurohypophysitis. There were several reports regarding the effectiveness of steroid therapy; therefore, the pharmacological dose of PSL was administered as first-line therapy, but the treatment did not reduce the hypothalamic lesion or prevent the emergence of Cushing’s syndrome. In 2008, treatment decisions were guided by pediatric LCH protocols, under which multifocal LCH or disease refractory to steroid therapy was treated with VBL, 6-MP, and PSL or with 2-CdA/cytarabine chemotherapy. As the second-line therapy, 2-CdA effectively reduced the hypothalamic lesion and ameliorated the peritumoral edema; however, the first cycle of 2-CdA (5 mg/body for 3 days) caused grade 4 bone marrow suppression. During the 2-month interval, and even after reducing the dose (4 mg/body for 3 days) for the second cycle, grade 4 bone marrow suppression recurred, leading to discontinuation of 2-CdA therapy. Although just two cycles of treatment significantly reduced the tumor on imaging, multiple anterior pituitary hormone deficiencies, CDI, hypothalamic dysfunction, and cognitive impairment were not alleviated. The therapeutic goals in hypothalamic–pituitary LCH are preventing infiltrative tissue damage and preserving endocrine function. However, even though surgery, radiotherapy, and chemotherapy have produced radiologic responses in some patients with tumorous lesions and, occasionally, neurodegenerative changes, established clinical signs and symptoms generally appear irreversible [5]; that is, chemotherapy for hypothalamic LCH does not prevent neurodegeneration or endocrinological dysfunction [1]. Therefore, optimizing quality of life and survival in hypothalamic LCH requires not only early tumor intervention to prevent neurodegeneration, but also rigorous, lifelong hormone replacement therapy, especially in cases with adipsic CDI.

## 4. Conclusions

We present a severe case of multisystem LCH complicated by hypothalamic–pituitary lesions. Although 2-CdA chemotherapy effectively reduced the lesion, irreversible hypothalamic dysfunction and endocrine disorders had occurred; the combination of adipsic CDI and cognitive impairment further complicated endocrine disorder management; ultimately, uncontrolled adipsic CDI resulted in fatal hypernatremia.

## Figures and Tables

**Table 1:** Endocrinological data at age 34 (in 2006).

Test Item	Data	Unit	Reference Range
TSH	2.23	mIU/L	0.35–3.73
Free triiodothyronine	2.65	pg/mL	2.2–4.1
Free thyroxine	0.83	ng/dL	0.90–1.80
LH	2.17	IU/L	1.71–8.59
FSH	5.84	IU/L	1.49–12.40
estradiol	10.4	pg/mL	28.8–196.8
ACTH	12.2	pg/mL	7.4–55.7
cortisol	20.0	μg/dL	8.0–25.0
GH	0.85	ng/mL	<2.10
IGF-1	141	ng/mL	115–277
PRL	78.2	ng/mL	4.3–13.7
ADH	0.2	pg/mL	0.0–3.1
Posm	298	mOsm/L	275–290

**Table 2:** Endocrinological data at age 35 (in 2007).

Test Item	Data	Unit	Reference Range
TSH	1.07	mIU/L	0.35–3.73
Free triiodothyronine	2.2	pg/mL	2.2–4.1
Free thyroxine	0.7	ng/dL	0.90–1.80
LH	<1.0	IU/L	1.71–8.59
FSH	1.84	IU/L	1.49–12.40
estradiol	<10.0	pg/mL	28.8–196.8
ACTH	5.4	pg/mL	7.4–55.7
cortisol	0.8	μg/dL	8.0–25.0
GH	0.2	ng/mL	<2.10
IGF-1	210	ng/mL	112–271
PRL	78.2	ng/mL	4.3–13.7

**Table 3:** A provocation test after intravenous injection of corticotropin-releasing hormone, thyrotropin-releasing hormone, gonadotropin-releasing hormone, and growth hormone-releasing factor demonstrated blunted responses in ACTH, LH, and FSH.

	0	15	30	60	90	120	Minutes
ACTH	5.4	9.8	12.6	13.7	13.9	9.8	pg/mL
GH	0.2	3.98	7.68	6.73	4.8	1.06	ng/mL
TSH	1.07	9.76	13.09	12.23	11.09	8.77	mIU/L
LH	<0.10	0.55	0.63	0.54	0.37	0.26	IU/L
FSH	1.84	5.9	7.55	8.75	8.83	8.29	IU/L
PRL	98.4	199.5	176.5	137.8	118.3	104.4	ng/mL

**Table 4:** Response of ADH during hypertonic saline test (5% NaCl). The hypertonic saline test showed no response in ADH.

	0	60	120	Reference Range
Posm	272	291	298	275–290
ADH	0.2	0.2	0.2	0.0–3.1

## Data Availability

Any data relevant to this case that are not presented in this manuscript can be obtained from the corresponding author upon reasonable request.

## Abbreviations

The following abbreviations are used in this manuscript:

ACTH	adrenocorticotropic hormone
ADH	antidiuretic hormone
APD	anterior pituitary dysfunction
CDI	central diabetes insipidus
CNS	central nervous system
CT	computed tomography
ddAVP	Desmopressin
FLAIR	fluid-attenuated inversion recovery
FSH	follicle-stimulating hormone
GH	growth hormone
HDS-R	Hasegawa’s dementia scale
HPS	hypothalamic–pituitary system
IGF-1	insulin-like growth factor 1
LCH	Langerhans cell histiocytosis
LH	luteinizing hormone
MRI	magnetic resonance imaging
Posm	plasma osmolar pressure
PRL	Prolactin
PSL	Prednisolone
TSH	thyroid-stimulating hormone
2-CdA	Cladribine
